# End-stage heart failure: Two surgical approaches with different rehabilitative outcomes

**DOI:** 10.1371/journal.pone.0185717

**Published:** 2017-10-03

**Authors:** Vittorio Racca, Paolo Castiglioni, Claudia Panzarino, Fabrizio Oliva, Enrico Perna, Maurizio Ferratini

**Affiliations:** 1 Cardiology Rehabilitation Center – Santa Maria Nascente Institute IRCCS, Don C. Gnocchi Foundation, Milan, Italy; 2 Biomedical Technology Department – Santa Maria Nascente Institute IRCCS, Don C. Gnocchi Foundation, Milan, Italy; 3 De Gasperis Cardio Center, Niguarda Hospital, Milan, Italy; Ospedale del Cuore G Pasquinucci Fondazione Toscana Gabriele Monasterio di Massa, ITALY

## Abstract

**Background:**

A rising number of patients are surgically treated for heart failure at the more advanced stage, thanks to the increasing use of left ventricular assist device (LVAD) as a reliable alternative to heart transplantation (HTx). However, it is still unknown whether differences exist between the two surgical approaches in the efficacy of rehabilitation programmes. Therefore, aim of this study was to evaluate whether functional capacity and rehabilitative outcomes differ between HTx and implantation of LVAD.

**Methods and results:**

We enrolled 51 patients with HTx and 46 with LVAD upon admission to our rehabilitation-unit. We evaluated six-minute walking test (6MWT), resting oxygen saturation (SaO_2_) and nutritional assessment before and after a standardised cardiovascular rehabilitation programme. HTx and LVAD groups differed in age, anthropometric variables, gender distribution. Upon enrolment, 6MWT distance was similar in the two groups, whereas malnutrition was less frequent and the waist circumference/height ratio (WHtR) was greater in LVAD patients. SaO_2_ was greater in HTx patients. Rehabilitation improved SaO_2_, 6MWT distance and nutritional status. The difference in malnutrition disappeared, but WHtR remained higher in the LVAD and SaO_2_ higher in the HTx patients; the 6MWT distance improved more in the HTx patients. Multivariate linear regression analysis confirmed that the type of intervention was independent predictor of 6MWT distance after rehabilitation.

**Conclusions:**

HTx patients improve more rapidly and perform better after rehabilitation, suggesting the need for more tailored rehabilitation training for LVAD patients.

## Introduction

Physicians are increasingly encountering patients with advanced, severe symptomatic heart failure (HF) in whom the mortality rate is high. Thanks to technological progress, heart transplantation (HTx) is no more the only surgical approach offering long-term outcome, and now nearly half of the patients are implanted with a left ventricular assist device (LVAD) as destination therapy [1]. One of the major challenges is to refer such patients promptly to heart transplant or LVAD implant centres before severe complications leading to irreversible organ dysfunction contraindicating surgical treatment. The International Society for Heart and Lung Transplantation (ISHLT) guidelines [2,3] help physicians to select HTx or LVAD on the basis of criteria that include age, anthropometric characteristics, various biological factors [4], and nutritional status because malnutrition may worsen clinical outcomes [5,6].

In general, end-stage HF patients who received HTx or LVAD implant should attend a cardiac rehabilitation programme to facilitate recovery after surgery. Such rehabilitation programmes consist in standardized sessions of physical exercises, with the same intensity and duration for HTx and LVAD implanted patients. However, the type of surgery, the criteria underlying its selection, as well as differences in therapies after surgery and in possible adverse events associated with each of the two therapies might affect the efficacy of cardiac rehabilitation differently in HTx and in LVAD patients. If this occurs, rehabilitation programmes better tailored to the type of surgical approach should be designed, so that the choice between LVAD and HTx does not affect recovery in patients with end-stage HF.

Therefore, aim of this observational study was to test the hypothesis that the rehabilitative outcome of a standardised cardiac rehabilitation programme, quantified as recovery in functional capacities and in nutritional status, differs between patients referred to our Rehabilitation Department after HTx or LVAD implantation.

## Methods

This open observational study was carried out at the Cardiology Rehabilitation Department of the Don Gnocchi Foundation’s Santa Maria Nascente Institute in Milan, Italy, in accordance with the principles of the Helsinki Declaration and Good Clinical Practice, and in observance of anti-discrimination regulations and standard privacy procedures. The study was approved by Ethics Committee of Don C. Gnocchi Foundation. S. Maria Nascente Institute IRCCS and all of the participants gave their written informed consent before entering the study.

Between 2014 and 2015, we consecutively enrolled all 97 adult patients admitted to our Cardiac Rehabilitation Unit as in-patients after HTx (n = 51) or the implantation of a LVAD with a continuous-flow pump (n = 46). Among the LVAD implanted patients, 31 (67%) received the Thoratec Heartmate II device and the remaining 15 (33%) the Heartware device. No selection criteria were used for referral or acceptance on the rehabilitation programme, and there were no exclusion criteria other than a refusal to give consent. All of the patients were white, and were directly transferred to our Institute on the day they were discharged from the Heart Transplantation Centre of Niguarda Hospital, the only heart transplantation centre in Milan.

After admission to the Cardiac Rehabilitation Unit, each patient underwent a complete cardiac assessment. The duration of HF symptoms was recorded, and the patients were classified as having acute HF (symptoms lasting for <1 month), subacute HF (symptoms lasting 1–12 months) or chronic HF (symptoms lasting for >12 months) at the time of surgery. We also recorded the degree of pre-surgical urgency in both groups as expressed by the Interagency Registry for Mechanically Assisted Circulatory Support (INTERMACS) scale [7], and in the HTx group also by United Network for Organ Sharing (UNOS) status [8].

Mean arterial blood pressure (MAP) in LVAD implanted patient was measured using the von Recklinghausen oscillotonometer (SK Speidel & Keller ALTERA), slowly inflating the rubber arm cuff to the level where the blood flow is absent and deflating until it reappears. In HTx patients systolic (SAP) and diastolic (DAP) arterial pressure were measured with a traditional sphygmomanometer and MAP was derived as (SAP+2×DAP)/3.

### Pharmacotherapy

All of the drugs taken by the patients were registered. The immunosuppressive regimen in the HTx patients (prednisolone, cyclosporine, tacrolimus, and mycophenolate mofetil or everolimus) was adjusted on the basis of laboratory tests and endomyocardial biopsy reports. Allograft rejection was diagnosed using the standardised grading system [9] in accordance with the ISHLT guidelines.

### Rehabilitation programme

The rehabilitation programme was the same for both groups, and was continued throughout the period of hospitalisation. In accordance with the European Association of Cardiovascular Prevention and Rehabilitation guidelines [10], it consisted of standardised physical training with incremental exercises supervised by expert physiotherapists; incentive spirometry; breathing exercises; sub-maximal incremental endurance training; and walking on a treadmill or cycling on an exercise bicycle. The sessions involved only aerobic exercises, lasted at least 30 minutes, and were carried out at least once a day on six days a week. Target heart rate, in bpm, was 70% the theoretical maximum heart rate, the latter calculated as difference between 220 and the patient’s age, in years (for instance, target heart rate in a 30 years old patient was (220–30)* 0.7 = 133 bpm). During the sessions, a safety protocol was followed as recommended for chronic HF patients undergoing cardiac rehabilitation [10]. The duration of the sessions was increased by ten minutes every three days if the patients’ cardiovascular conditions permitted, until it reached a maximum of 50 minutes twice a day, and there was a gradual increase in resistance. The increases were made cautiously in order to avoid the occurrence of musculoskeletal or cardiorespiratory complications during training, particularly in the LVAD patients as exertion can increase right ventricular failure. No adaptation of LVAD was made during exercise training. The programme was interrupted during graft rejection episodes in the HTx patients. This happened with a low incidence, in 2 out of 51 patients (4%), causing interruptions, never lasting more than two weeks. The LVAD group rehabilitative programme was interrupted in 4 out of 46 patients (8.7%) because of ventricular arrhythmia (3 cases) or severe anemia (1 case). Twelve other patients in the LVAD group (26.1%) had minor infective or arrhythmic complications, not interfering with the rehabilitative programme.

The activities included limb flexion, extension and abduction, neck flexion and extension, and trunk flexion, extension and rotation. When necessary, physical therapy aimed at improving balance was included in order to obtain independent functional mobility. Heart rate and oxygen saturation were telemetrically monitored while the patients were exercising, and blood pressure was measured manually at the beginning and end of each session. Patient compliance with the training protocol was evaluated by the physiotherapist and recorded in a daily report. Between sessions, the patients were allowed to walk in the rehabilitation unit and carry out their everyday activities.

The planned duration of the rehabilitation programme was three weeks, but the actual timing of discharge was based on the stability of the patients’ clinical condition; the absence of clinical complications, infections, or graft rejection; and the satisfactory achievement of goals such as improved aerobic fitness, psychosocial well-being, increased participation in occupational and recreational activities, and improvements in opportunities for independent self-care. All these targets can be considered as indexes of the beneficial impact of the rehabilitation programme. In our study, we considered the cardiac rehabilitation outcome as composed by the improvements in distance walked during the 6MWT, in resting oxygen saturation, in peak workload and in nutritional status.

### Nutritional and functional assessments

Body weight and height were assessed upon admission (T1) and at the end of the rehabilitation (T2), and the body mass index (BMI) was calculated as the ratio between weight and height squared (in kg/m^2^). Waist circumference was measured at the level of the umbilical line at T1 and T2 keeping the tape on the horizontal plane; and the ratio between waist circumference and height (WHtR) was calculated. The patients’ WHtR was classified as *low* (<0.4), *normal* (≥0.4 but <0.5), *high* (≥0.5 but <0.6) or *very high* (≥0.6) according to Ashwell *et al*. [11]. A trained nurse evaluated nutritional risk at T1 and T2 using the Malnutrition Universal Screening Tool (MUST) [12]: the patients were considered as being at high risk of malnourishment if they had a MUST score of ≥2.

In accordance with the guidelines of the American Thoracic Society [13], the patients underwent a 6-minute walk test (6MWT) at T1 and T2, and resting oxygen saturation (SaO_2_) was non-invasively measured at the same times: SaO2 was measured at the finger by the Mindray PM-60 infrared pulse oxymeter device.

### Statistical analysis

Non-parametric tests were used in order to avoid making assumptions about the statistical distribution of the data. The quantitative variables were compared between the HTx and LVAD groups using the Mann-Whitney U test, and between T1 and T2 using Wilcoxon’s matched pairs test. The differences in the distribution of categorical variables between the HTx and LVAD groups were tested using the chi-squared test and, when they were significant, the statistical significance of the difference was evaluated separately for each class of categorical variable using Fisher’s exact test. All of the tests were two tailed, and p values of <0.05 were considered statistically significant.

Multivariate linear regression analysis was used to identify the independent predictors of the main rehabilitative outcome, the distance walked during the 6MWT at T2 as the dependent variable. The predictors were the type of surgery (LVAD = 0 and HTx = 1), age, gender (male = 0, female = 1), the duration of rehabilitation, and BMI and WHtR at T2 (body weight and waist circumference were not included in the multivariate model because they appear directly as the numerator in the formulae used to calculate BMI and WHtR).

## Results

### Differences upon admission

**Fig 1:**
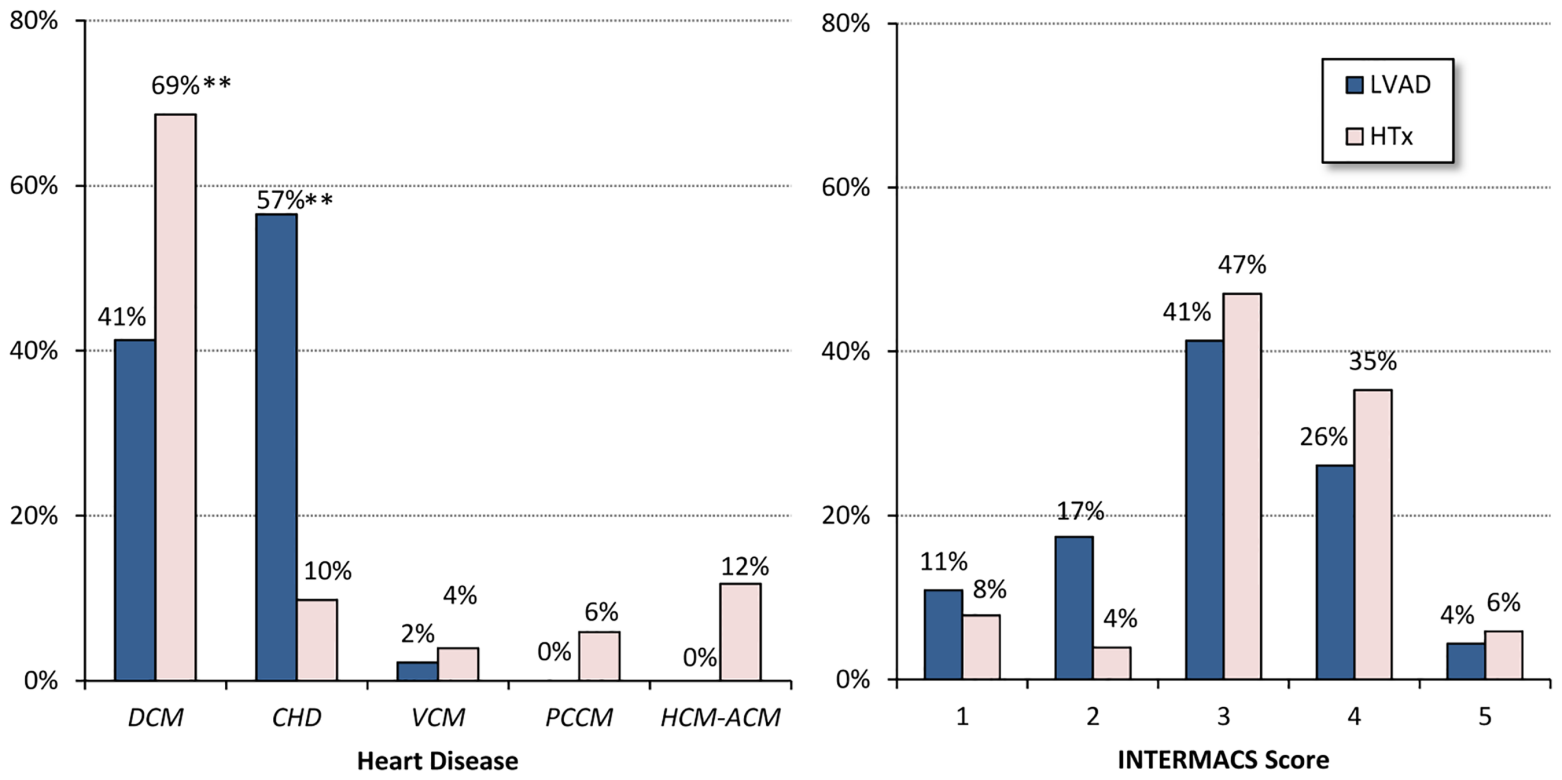
Type and severity of heart disease. *Left*: Prevalence of heart disease leading to end stage heart failure (HF), separately for patients surgically treated with LVAD implant or Heart Transplantation (HTx). DCM = Dilated Cardiomyopathy; CHD = post-ischemic Cardiomyopathy; VCM = Valvular Cardiomyopathy; PCCM = Postchemotherapy Cardiomyopathy; HCM-ACM = Hypertrophic or Arrythmogenic Cardiomyopathy; LVAD and HTx distributions differ significantly (p<0.001, Chi-square test); the ** indicates differences between groups significant at p<0.01 (Fisher’s exact test). *Right*: severity of heart disease expressed as INTERMACS score; distributions do not differ between groups (p = 0.24,Chi square test).

The more frequent cardiac disease leading to end-stage HF was post-ischemic cardiomyopathy in the LVAD patients and dilated cardiomyopathy in the HTx patients (Fig 1, left). The severity of end-stage HF was high or very high in most of the patients, and there was no between-group difference in the distribution of the INTERMACS scores [7] (Fig 1, right). The UNOS Status classification [8] confirmed the severity of the disease, with most of the HTx patients being classified as 1A (41%) or 1B (39%), and only 20% as 2A.

Disease duration before heart surgery was similar in LVAD and HTx groups (8.1 ± 8.0 *vs* 8.9 ± 7.5 years). Most of the patients were affected by chronic HF (LVAD: 80.5%; HTx: 86.3%); 15.2% of LVAD and 7.8% of HTx patients had subacute HF; and 4.3% of LVAD and 5.9% of HTx patients had acute HF.

Table 1 summarises the general characteristics of the patients (see S1 Table for comorbidities, and S2 Table for the prescribed heart failure therapies, in the two groups separately). The patients were admitted to the Cardiac Rehabilitation Unit 30.9 ±16.1 days (mean ±SD) after surgery, with a longer time interval of about one week in the LVAD group (Table 1). Most of the patients were males, who were prevalent in the LVAD group (89.1% *vs* 60.8%). The LVAD patients were older and their mean BMI, which was within the normal range in both groups, was significantly higher; they also had a significantly larger waist circumference and WHtR, and tended to be taller (Table 1).

**Table 1:** General characteristics and prevalence of nutritional risk factors in LVAD implanted and HTx patients, with significance p of the difference between groups.

	Admission (T1)			End of Rehabilitation (T2)		
	LVAD (N = 46)	HTx (N = 51)	p	LVAD (N = 46)	HTx (N = 51)	p
*Sex (M/F)*	41/5	30/21	<0.01	-	-	-
*Age (yrs)*	57.3 (7.8)	48.0 (13.6)	<0.01	-	-	-
*Height (cm)*	1.73 (0.08)	1.69 (0.09)	0.05	-	-	-
*BMI (kg/m*^*2*^*)*	25.3 (4)	22.2 (3.8)	<0.001	25.2 (3.3)	22.0 (3.2)	<0.001
*Waist (cm)*	100.2 (10.6)	89.1 (11.9)	<0.001	99.3 (9.5)	88.0 (10.5)	<0.001
*WHtR*	0.58 (0.06)	0.53 (0.06)	<0.001	0.58 (0.06)	0.52 (0.05)	<0.001
*Time from Surgery (days)*	34.6 (16.8)	27.7 (14.9)	<0.05	68.8 (24.3)	67.4 (23.2)	0.77
*BNP (ng/mL)*	428 (455)	435 (284)	0.95	325 (243)	391 (395)	0.69
*Comorbidities* ^1^						
*Number per patient*	1.7 (1.2)	1.1 (0.9)	<0.01			
*Patients without comorbidities*	17.4%	25.5%	0.47			
*History of hypertension*	15.2%	5.9%	0.18			
*Nutritonal Risk Factors*						
*Malnutrition*	0%	9.8%	0.06	0%	7.8%	0.12
*Obesity (stage 1)*	15.2%	3.9%	0.08	10.9%	2.0%	0.098
*Abdominal Obesity*	41.3%	23.5%	0.08	37.0%	21.6%	0.12
*Metabolic Syndrome*	23.9%	11.8%	0.18	20.6%	9.8%	0.21

Anthropometric indices as mean (sd): p after Mann Whitney U test; prevalence of risk factors (percentage) and sex: p after Fisher’s exact test. Obesity stage 1 defined by BMI ≥ 30.0 Kg/m^2^. Abdominal obesity defined by waist circumference >88 cm in female or >102 cm in male. Metabolic syndrome defined as abdominal obesity and at least two of the following: glucose >100 mg/dL, Triacylglycerol > 150 mg/dL, HDL Cholesterol < 40 mg/dL in male or < 50 mg/dL in female, blood pressure ≥ 130/85 mmHg;

^*1*^ see S1 Table for a complete list of comorbidities.

BMI = body mass index; WHtR = waist/height ratio; BNP = brain natriuretic peptide.

All of the anthropometric characteristics except for height were substantially different between LVAD and HTx patients even when evaluated separately by sex (Table 2).

**Table 2:** Baseline characteristics by sex of patients in LVAD implanted and HTx groups, as mean (SD).

	Males				Females			
	LVAD (N = 41)	HTx (N = 30)	Δ%	p	LVAD (N = 5)	HTx (N = 21)	Δ%	p
*Age (yrs)*	56.8 (7.6)	47.4 (12.9)	19.8%	<0.01	61.4 (9.4)	49.0 (15.0)	23.7%	0.19
*BMI (kg/m*^*2*^*)*	25.3 (3.9)	22.5 (3.8)	12.2%	<0.01	26.1 (5.1)	21.6 (3.7)	20.4%	0.05
*Height (cm)*	174.0 (7.0)	173.9 (6.0)	0.1%	0.74	164.2 (7.0)	161.3 (7.3)	1.8%	0.67
*Waist (cm)*	100.4 (10.5)	91.1 (10.7)	10.3%	<0.01	97.8 (12.9)	86.2 (13.3)	13.5%	0.07
*WHtR*	0.58 (0.06)	0.52 (0.06)	10.3%	<0.01	0.60 (0.09)	0.53 (0.07)	12.0%	0.09

Measures at baseline (T1); **Δ%** is the difference between LVAD and HTx values as percentage of the HTx value; p after Mann Whitney U test.

**Fig 2:**
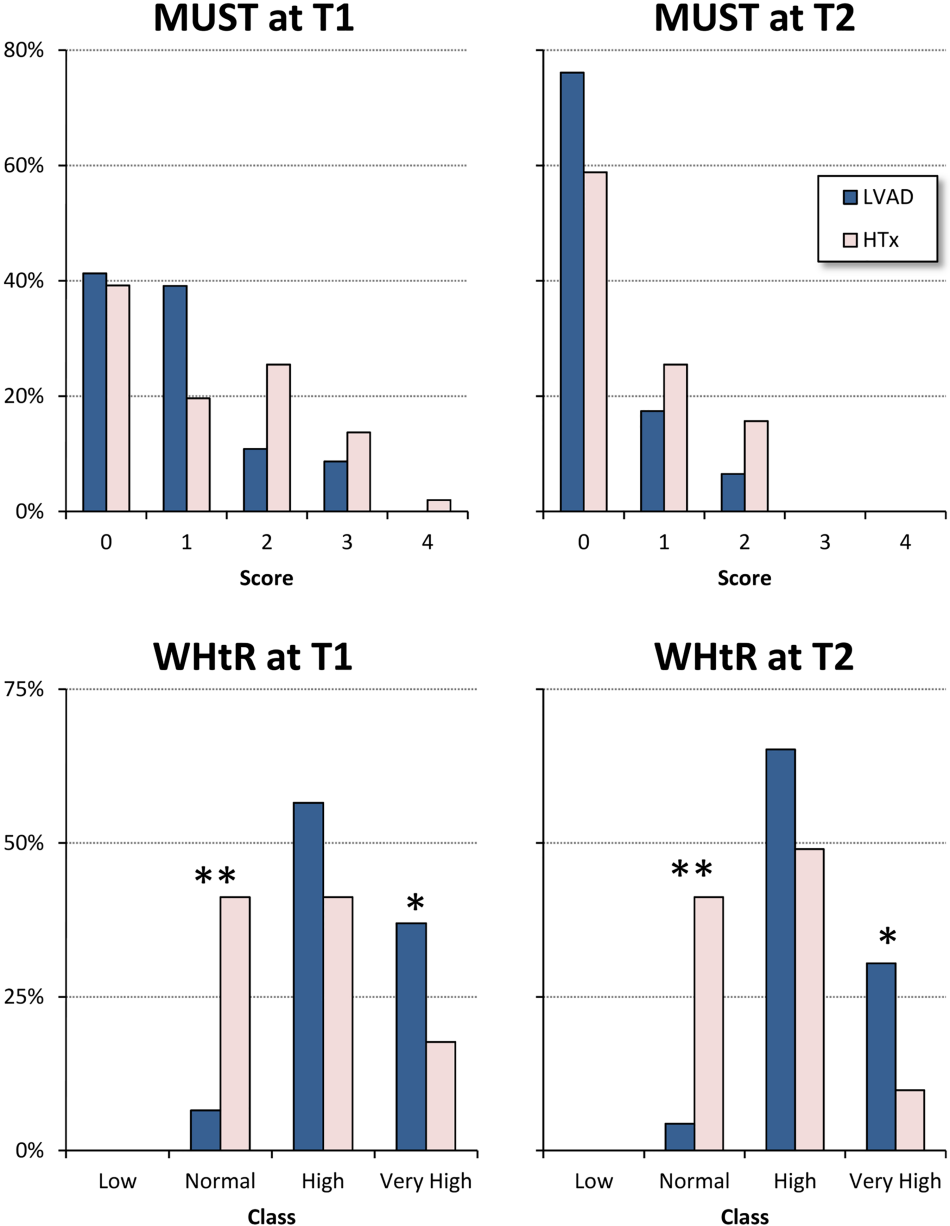
Nutritional status in LVAD implanted and HTx patients, at admission (T1) and end of rehabilitation (T2). *Upper panels*: distributions of Malnutrition Universal Screening Tool (MUST) scores: distributions do not differ significantly between HTx and LVAD groups neither at T1 nor at T2 (p = 0.11 and = 0.17 respectively, after Chi-square test). *Lower panels*: prevalence of each class of Weight-to-Height ratio (WHtR); distributions differed significantly between HTx and LVAD groups both at T1 and at T2 (p<0.001 after Chi-square test); the * and ** indicate differences between groups for a given WHtR class significant at p<0.05 and p<0.01 (Fisher’s exact test).

None of the patients in either group was underweight (BMI <18.5), but the prevalence of first-stage obesity (BMI ≥30 but <35) tended to be higher in the LVAD group (Table 1); none of the patients had stage 2 (BMI ≥35 but <40) or stage 3 obesity (BMI >40). Admission MUST scores indicating a high risk of malnutrition (≥2, see Fig 2, upper panels) were more frequent in the HTx group (41% *vs* 20%, p = 0.03). None of the patients had a low WHtR (Fig 2, lower panels), but a normal WHtR was significantly more frequent in the HTx group, and a very high WHtR was significantly more frequent in the LVAD group (Fig 2).

**Fig 3:**
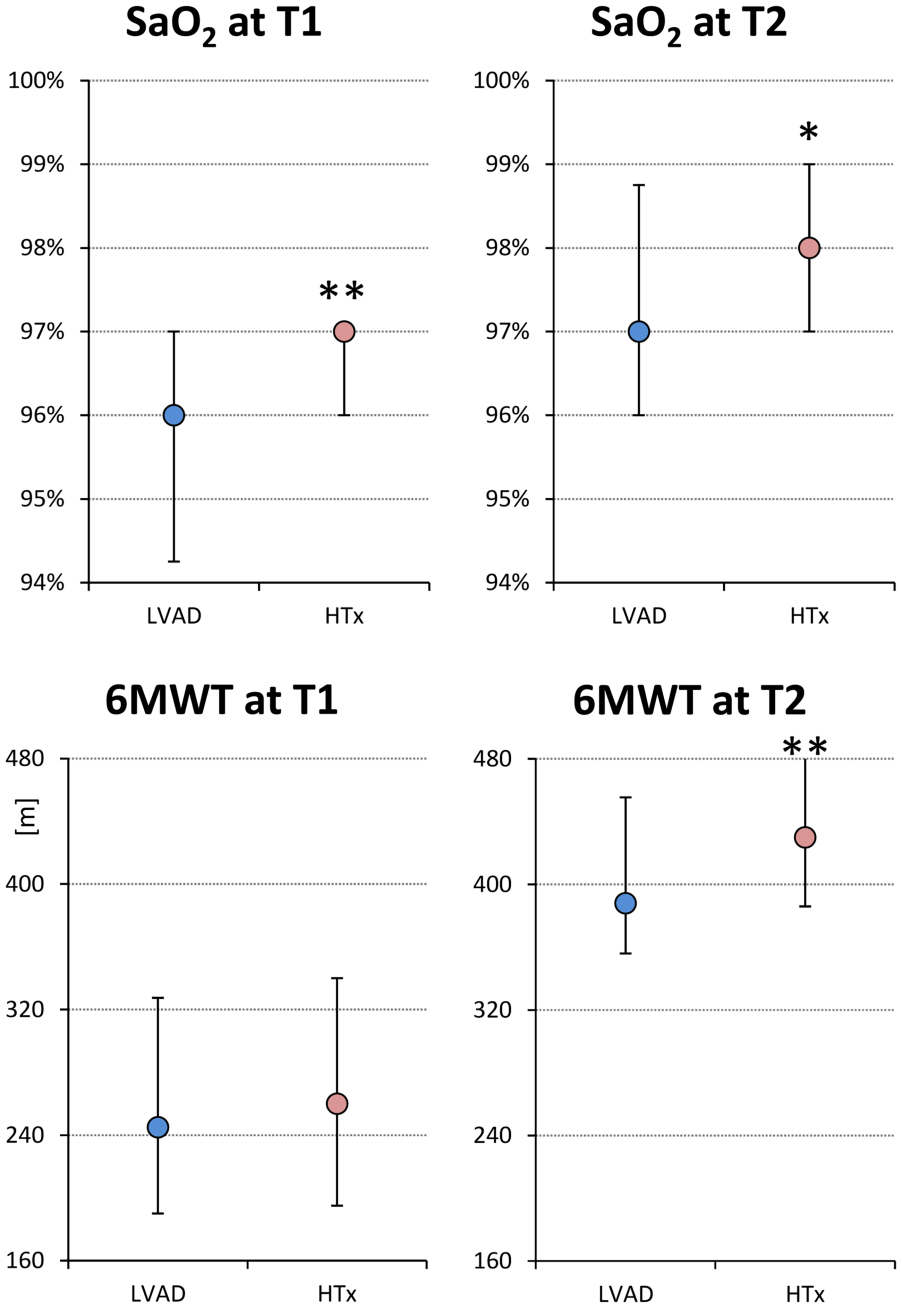
Oxygen saturation at rest (upper panels) and distance walked in 6 minutes (lower panels) in LVAD implanted and HTx patients, at admission (T1) and end of rehabilitation (T2). Values as median and quartiles. The ** indicates differences between groups significant at p<0.01 (Mann Whitney U test).

In terms of functional capacity upon admission, the HTx patients had significantly better resting SaO_2_ values (Fig 3, upper left panel). The 6MWT distance was similar in both groups (Fig 3, lower left panel), even when separating males (LVAD: 244 ±92 m; HTx: 281 ±121 m; p = 0.15) and females (LVAD: 219 ±113 m; HTx: 211 ±95 m; p = 0.82). The achieved heart rate at the peak of six minute walk test exercise was similar in the LVAD and HTx patients: 99 ±12 vs 97±11 bpm (p = 0.50).

At the beginning of the rehabilitation programme, baseline heart rate was slightly lower in the LVAD than in the HTx group (83 ±11 vs 89 ±10 bpm, p = 0.02). Similarly, the heart rate achieved at the end of exercise was slightly lower in the LVAD than in the HTx group (88±10 vs 95±12 bpm, p = 0.02). Both baseline MAP (LVAD: 86.4 ±5.7 mmHg; HTx: 89.2 ±8.8 mmHg; p = 0.09) and MAP at the end of exercise (LVAD: 86.1 ±6.3 mmHg; HTx: 89.5 ±9.1 mmHg; p = 0.23) were similar in the two groups. Also the peak workload was similar in the two groups of patients (LVAD: 5 ±8 Watts; HTx: 4 ±7 Watts, p = 0.45).

### Differences after rehabilitation

The average in-hospital stay was 37.2 ±16.7 days, with no significant difference between the HTx (39.7 ±17.4) and LVAD patients (34.2 ±15.8, p = 0.12). The rehabilitation session attendance and completion rate was similarly high: 76% in the HTx group and 82% in the LVAD group. The percentage of patients with a MUST score of ≥2 significantly decreased in both groups (p<0.01), with no significant difference between the HTx (16%) and LVAD group (7%) (Fig 2). Moreover, no patients had a score equal to 3 or 4. Furthermore, the rehabilitation programme significantly decreased waist circumference and WHtR in the group, with similar changes in both groups (Table 3).

**Table 3:** Changes between T2 and T1 as mean (SD), in the whole group and separately in LVAD and HTx patients.

	All (N = 97) Δ T2-T1	p ^a^ T2 vs T1	LVAD (N = 46) ΔT2-T1	HTx (N = 51) Δ T2-T1	p ^b^ LVAD vs HTx
*Weight (kg)*	-0.65 (3.36)	0.07	-0.67 (3.66)	-0.63 (3.10)	0.88
*BMI (kg/m*^*2*^*)*	-0.2 (1.2)	0.12	-0.2 (1.4)	-0.2 (1.1)	0.81
*Waist (cm)*	-0.96 (2.72)	<0.01	-0.80 (2.35)	-1.10 (3.02)	0.68
*WHtR*	-0.005 (0.016)	<0.01	-0.004 (0.015)	-0.006 (0.018)	0.51
*SaO*_*2*_ *(%)*	0.48 (2.39)	<0.05	0.48 (2.81)	0.49 (1.95)	0.71
*6MWT (m)*	154 (109)	<0.01	129 (114)	177 (100)	<0.05

^a^ significance p of the difference between T2 and T1 conditions by Wilcoxon’s test;

^b^ significance p of the difference between LVAD and HTx groups by Mann-Whitney test.

The rehabilitation programme also significantly increased resting SaO_2_, with similar increases in both groups (Table 3); the value at the end of rehabilitation therefore remained higher in the HTx group (Fig 3, right).

After rehabilitation, baseline heart rate was slightly lower in the LVAD than in the HTx group (81 ±9 vs 86 ±10 bpm, p = 0.03); however, the difference in heart rate at the end of exercise was not significant (91±9 vs 95±12 bpm, p = 0.08). Baseline MAP (LVAD: 85.8 ±7.2 mmHg; HTx: 85.2 ±9.2 mmHg; p = 0.73) and MAP at the end of exercise (LVAD: 85.7 ±6.2 mmHg; HTx: 86.5 ±7.1 mmHg; p = 0.64) were similar in the two groups. The peak workload was also similar (LVAD: 23 ± 18 watts; HTx: 26 ± 91 watts, p = 0.50).

As expected, rehabilitation substantially increased the distance covered during the 6MWT (Table 3). The improvement was greater in the HTx group (Table 3), whose patients walked a significantly longer distance at T2 than those in the LVAD group (Fig 3), without any difference in achieved heart rate: 102±12 vs 103±12 bpm (p = 0.52).

Multivariate analysis indicated that the type of surgery was a significant independent predictor of 6MWT performance at T2; the beta value of 0.26 indicates that the HTx patients walked further than the LVAD patients. Age also proved to be a statistically significant independent predictor; the beta value of -0.26 indicates that the younger patients walked further than their older counterparts. Gender was only marginally significant (p = 0.07); the beta value of -0.18 indicates that males walked further than females. BMI (p = 0.83, beta = 0.04), WHtR (p = 0.44, beta = -0.14) and the duration of rehabilitation (p = 0.44, beta = -0.14) were not independent predictors of 6MWT performance at T2.

## Discussion

This study compares patients who have undergone a HTx with those who have received the implantation of an LVAD at the time of their discharge from a cardiac surgery unit and after completing the same cardiac rehabilitation programme. Upon admission to our Rehabilitation Unit, the groups differed in terms of anthropometric variables, gender distribution, nutritional status and comorbidities prevalence. Malnutrition was more frequent among the HTx patients (who also had better SaO_2_ values), and a very high WHtR was more frequent among the LVAD patients. At the end of the cardiac rehabilitation period, nutritional status had improved and there was no longer any difference in the malnutrition score; however, there was still a between-group difference in the WHtR. Rehabilitation also improved resting SaO_2_ and the 6MWT distance, but the HTx patients walked longer distances and still had better SaO_2_ values. Although the differences in age and gender distribution between the groups may contribute to explaining the greater distance walked by the HTx patients after rehabilitation, the type of surgery (HTx or LVAD) remained an independent predictor.

All of the patients were selected and treated at the same transplantation centre, and the duration of HF symptoms and pre-surgical urgency were similar in the two groups. It is therefore unlikely that differences in the duration or severity of HF symptoms were responsible for different rehabilitative outcomes. However, the HTx patients were discharged from the transplant centre about one week earlier, suggesting better post-surgical recovery than in the LVAD group.

The prevalence of the underlying cardiac diseases was surprisingly different between the groups considering that in most of the patients LVAD implantation was proposed as a bridge to transplant and only one had a clear contraindication because older than the limit laid down in the international guidelines. In practice, however, the shortage of heart donors in front of the increasing use and reliability of these devices implies that the majority of LVAD implanted patients will not receive heart transplantation. This aspect should be taken into account by heart surgeons when they candidate a patient to LVAD implant. The higher incidence of idiopathic cardiomyopathy in the HTx patients may be explained by their younger age or the lower risk of mortality in patients who receive a transplant because of idiopathic cardiomiopathy than in those undergoing transplantation because of coronary artery disease [14]. Ischemic heart disease was prevalent among the LVAD patients, probably because of their older age. None of the LVAD patients had hypertrophic or arrhythmogenic cardiomyopathy because an LVAD is not appropriate for small ventricular cavities or patients with a normal ejection fraction.

The age difference between the groups was probably due to the priority given to younger HTx candidates: the mean age of our HTx patients corresponds to that reported by the ISHLT in relation to a large HTx patient population [14]. The low prevalence of females among our LVAD patients was probably due to negative selection prompted by smaller BMI of females. Except for height, the anthropometric differences between the two groups were not due to the unequal gender distribution, but probably to the same selection criteria, whereas the gender-related difference in height may have been due the worse prognosis of HTx in the case of a negative height mismatch: incidence of coronary allograft vasculopathy within eight years is higher when the donor is 5 cm smaller than the recipient [8].

The nutritional status of our patients upon admission to rehabilitation was better than that reported by others [15], probably because of the preventive nutritional care plan used by the transplant centre. BMI may be misleading in decompensated patients with marked fluid retention, but none of our patients had edema at the time they were admitted to our unit, and the calculations indicated that none of the HTx patients was affected by stage 2 or 3 obesity. A BMI of >35 is an established contraindication to HTx as a high pre-transplant BMI is associated with worse outcomes [2] and consequently weight loss is strongly recommended before putting obese patients on the waiting for cardiac transplantation. Stage 1 obesity was more prevalent in our LVAD patients, but none of them was in a higher stage probably because of the hypocaloric diet prescribed to achieve a BMI of ≤35 kg/m^2^ as extreme obesity is associated with a high risk of death after LVAD implantation [16]. Moreover, few of the LVAD patients were at risk of malnutrition, probably because the procedure selection criteria took into account the fact that a poor nutritional status before implantation is associated with re-hospitalisation, a worse prognosis, and increased mortality [17,18].

Despite the shorter time between the surgical operation and admission to rehabilitation, the HTx patients had better SaO_2_ than the LVAD patients, probably favoured by a faster recovery. This is also suggested by their performance in the 6MWT, similar to the LVAD group, if one considers that HTx patients were smaller than LVAD patients.

Rehabilitation decreased waist circumference and the WHtR, but not weight or the BMI, thus suggesting that visceral fat decreased and peripheral muscle mass increased. After rehabilitation, the HTx patients had better resting SaO_2_ values and performed better at the 6MWT. The fact that the distance walked remained significantly longer in the HTx group even after correcting for age, gender, nutritional status and the duration of rehabilitation confirms the view that HTx is still the best option.

Patients with refractory, advanced HF may be selected to undergo HTx or the implantation of a LVAD on the basis of a complex decision making process that involves their clinical condition, their different prevalence of comorbidities, the anthropometric characteristics and the nutritional status. All of these factors may have contributed to the finding that HTx patients recover more rapidly than LVAD patients, which we report for the first time. However, the functional performance of our LVAD patients also substantially increased after rehabilitation and, as LVAD technology is improving, in the next future all patients eligible for HTx, with risk of rapid deterioration and with low probability to receive a donor heart in time, will be likely referred to this procedure [19].

Our results would suggest the need of a tailored rehabilitation training for the increasing population of LVAD implanted patients, such as a more careful and personalized nutritional intervention and a longer period of rehabilitation due to their slower recovery.

### Limitations

Patients were treated in a single cardiac transplant centre, and selection criteria of other centers might differ. Moreover, in Italy a 3-week cardiac rehabilitation program is offered free of charge by National Health System to all patients after heart transplant or LVAD implant. Thus, it was not possible to compare patients who received with those who did not receive cardiac rehabilitation to separately quantify the effects of cardiac rehabilitation from the effects of a 3-week recovery period after surgery.

## Supporting information

S1 Table List of comorbidities in L-VAD implanted and HTx patients. Values as N (%).
(DOCX)

S2 Table Heart Failure therapy in L-VAD implanted and HTx patients. Values as N (%).
(DOCX)
