# Supplementary material for: End-stage heart failure: Two surgical approaches with different rehabilitative outcomes
Source: PLoS One. 2017 Oct 3;12(10):e0185717. doi: 10.1371/journal.pone.0185717 (PMC5626463; doi:10.1371/journal.pone.0185717)
Supplement: S1 Table — Values as N (%). (DOCX) [file pone.0185717.s001.docx]

**S1 Table. List of comorbidities in L-VAD implanted and HTx patients.** Values as N (%)

|  | **L-VAD (N=46)** | **HTx (N=51)** |
| --- | --- | --- |
| Chronic Pulmonary Disease^1^ | 11 (23.9) | 5 (9.8) |
| Obstructive Sleep Apnea Syndrome | 3 (6.5) | 2 (3.9) |
| Chronic Renal Failure | 5 (10.9) | 4 (7.8) |
| Diabetes Mellitus | 12 (26.1) | 3 (5.9) |
| Cerebrovascular accident | 2 (4.35) | 3 (5.9) |
| Peripheral Vascular Disease | 2 (4.35) | 1 (2.0) |
| Abdominal Aortic Aneurysm | 1 (2.17) | 0 (0.0) |
| Non vascular neurologic disorders | 2 (4.35) | 1 (2.0) |
| Thrombocytopenia | 1 (2.17) | 1 (2.0) |
| Obesity (stage 1 or 2) | 8 (17.4) | 2 (3.9) |
| Chronic Hepatitis B or C | 2 (4.35) | 0 (0.0) |
| Lymphoma | 0 (0.0) | 2 (3.9) |
| MGUS ^2^ | 0 (0.0) | 1 (2.0) |
| History of breast cancer | 0 (0.0) | 2 (3.9) |
| History of bladder papillary tumor | 1 (2.17) | 0 (0.0) |
| Hypothyroidism | 6 (13.4) | 12 (23.5) |
| Hyperthyroidism | 1 (2.17) | 8 (15.7) |
| Adrenal adenoma | 1 (2.17) | 2 (3.9) |
| Hypophyseal adenoma | 1 (2.17) | 0 (0.0) |
| Acromegaly | 2 (4.35) | 0 (0.0) |
| Coxartrosis | 0 (0.0) | 1 (2.0) |
| Renal infarction | 0 (0.0) | 1 (2.0) |
| Nephrolithiasis | 1 (2.17) | 0 (0.0) |
| Benign Prostatic Hyperplasia | 3 (6.52) | 0 (0.0) |
| Colon Polyps | 5 (10.9) | 2 (3.9) |
| Diverticulitis colon | 1 (2.17) | 0 (0.0) |
| Colelitiasis | 1 (2.17) | 1 (2.0) |
| Gastro-esophageal reflux disease | 1 (2.17) | 0 (0.0) |
| Chronic peptic ulcer disease | 2 (4.35) | 0 (0.0) |
| Hyatal hernia | 2 (4.35) | 0 (0.0) |
| Gout | 0 (0.0) | 1 (2.0) |
| Sarcoidosis | 1 (2.17) | 0 (0.0) |

^1^ obstructive or restrictive; ^2^ monoclonal gammopathy of undetermined significance.
