# Supplementary material for: End-stage heart failure: Two surgical approaches with different rehabilitative outcomes
Source: PLoS One. 2017 Oct 3;12(10):e0185717. doi: 10.1371/journal.pone.0185717 (PMC5626463; doi:10.1371/journal.pone.0185717)
Supplement: S2 Table — Values as N (%). (DOCX) [file pone.0185717.s002.docx]

**S2 Table. Heart Failure therapy in L-VAD implanted and HTx patients.** Values as N (%).

|  | **L-VAD (N=46)** | **HTx (N=51)** | **p** |
| --- | --- | --- | --- |
| Beta blockers | 31 (67.4) | 19 (37.2) | <0.01 |
| ACE-inhibitors | 31 (67.4) | 32 (62.7) | 0.79 |
| Angiotensin receptor blockers | 3 (6.5) | 2 (3.92 | 0.90 |
| Loop diuretics (Furosemide) | 31 (67.4) | 25 (49.0) | 0.10 |
| Aldosterone antagonists | 18 (39.1) | 1 (2.0) | <0.0001 |
| Cardiac glycosides (Digoxin) | 4 (8.7) | 0 (0.0) | 0.10 |
| Amiodarone | 21 (46.6) | 1 (2.0) | <0.0001 |

p after chi-squared test
